# Supplementary material for: Do we need medical imaging-informed musculoskeletal models for simulations in healthy adults? A new workflow based on magnetic resonance imaging highlights the importance of personalized geometry
Source: PLoS Comput Biol. 2026 Mar 16;22(3):e1014073. doi: 10.1371/journal.pcbi.1014073 (PMC13029800; doi:10.1371/journal.pcbi.1014073)
Supplement: S1 Appendix — (PDF) [file pcbi.1014073.s001.pdf]

Do We Need Medical Imaging-Informed Musculoskeletal Models for Simulations in Healthy Adults? A New Workflow Based on Magnetic Resonance Imaging Highlights the Importance of Personalized Geometry

Ekaterina Stansfield[1], Willi Koller[2], Basílio Gonçalves[2], and Hans Kainz[2]

[1]Department of Evolutionary Biology, University of Vienna, Austria
[2]Department of Sport and Human Movement Science, University of Vienna, Austria

S1 Appendix

Index

1 Supplement to Results 2
2 Main Workflow 3
3 Muscle Paths and Joint Reaction Forces Comparison 6
4 Model Validation 9
5 Muscle Activations and EMG Data 10

1 SUPPLEMENT TO RESULTS

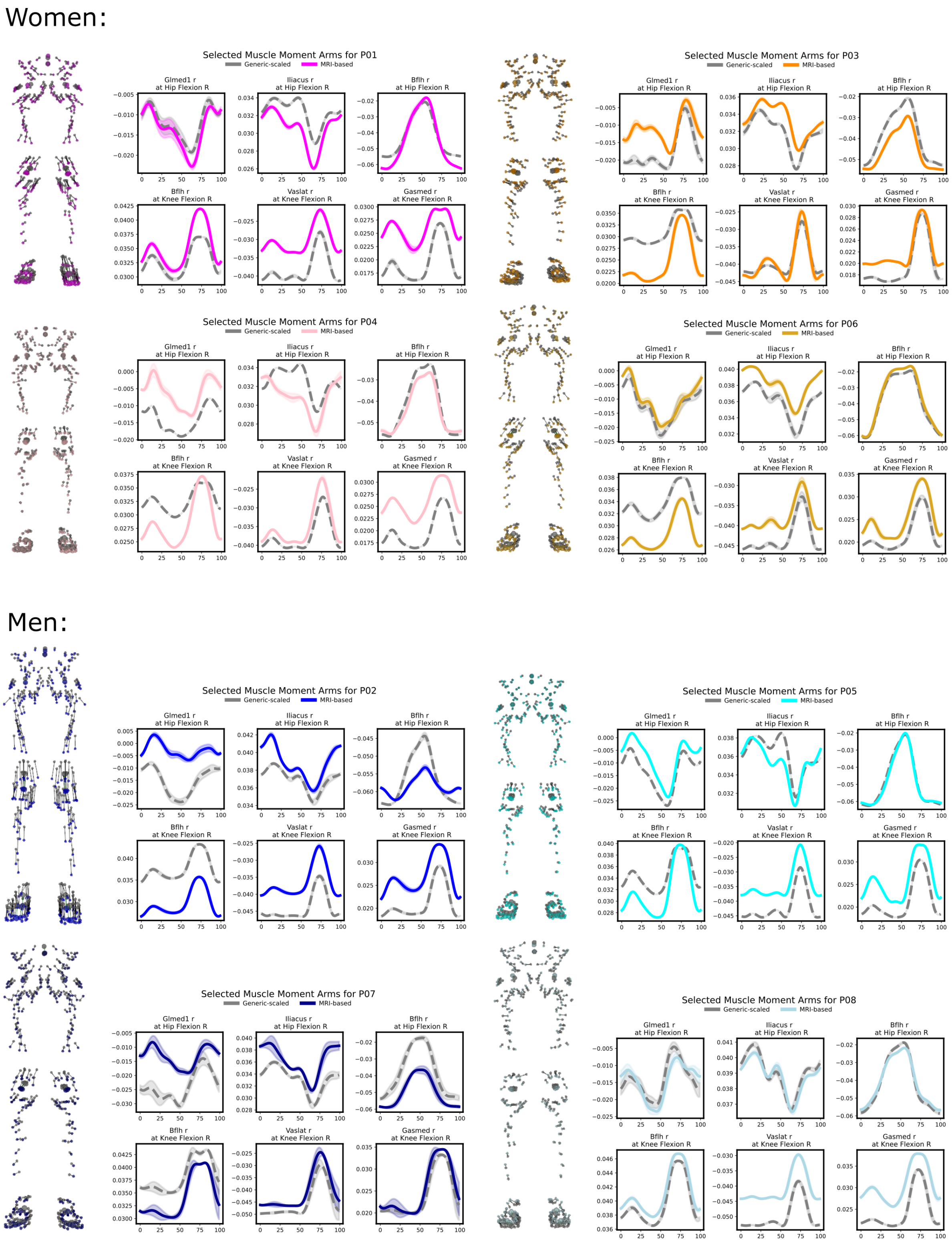

Figure A. Superposition of MRI-based and Generic-scaled joint centers and muscle paths in ground frame and the resulting differences moment arms of a chosen set of muscles.

2 MAIN WORKFLOW

Table A. Names and descriptions of skin markers.

Marker	Description	Marker	Description
C7	7th cervical vertebra	LTH3	Left Thigh tracking marker 3
RBAK	right back	LGT	Left Greater Trochanter
CLAV	clavicle, jugular notch	LKNE	Left Knee, lateral
STRN	sternum	LMKNE	Left Medial Knee
T10	10th toracic vertebra	RTB1	Right Tibia tracking marker 1
RASI	Right Anterior Superior Iliac Spine	RTB2	Right Tibia tracking marker 2
RPSI	Right Posterior Superior Iliac Spine	RTB3	Right Tibia tracking marker 3
LPSI	Left Posterior Superior Iliac Spine	RANK	Right Ankle, lateral
LASI	Left Anterior Superior Iliac Spine	RMMA	Right medial Malleolus
PE01	Sacrum tracking marker 1	LTB1	Left Tibia tracking marker 1
PE02	Sacrum tracking marker 2	LTB2	Left Tibia tracking marker 2
PE03	Sacrum tracking marker 3	LTB3	Left Tibia tracking marker 3
RTH1	Right thigh tracking marker 1	LANK	Left Ankle, lateral
RTH2	Right thigh tracking marker 2	LMMA	Left Medial Malleolus
RTH3	Right thigh tracking marker 3	RHEE	Right Heel
RGT	Right Greater Trochanter	RD5M	Right 5th Digit Medial
RKNE	Right Knee, lateral	RTOE	Right Toe
RMKNE	Right Medial Knee	LHEE	Left Heel
LTH1	Left Thigh tracking marker 1	LD5M	Left 5th Digit Medial
LTH2	Left Thigh tracking marker 2	LTOE	Left Toe

Table B. Definitions of scaling dimensions. Marker pairs are given without a comma. The comma separates pairs whose mean was used for scaling.

Dimension name	Markers	Segments	Scaled axes
pelvis width	LASI RASI, LPSI RPSI	pelvis	X Z
pelvis height	LASI LMKNE, RASI RMKNE	pelvis	Y
torso height	PE01 C7	torso	Y Z
torso depth	T10 STRN	torso	Z
femur_r_length	RASI RMKNE, RGT RKNE	femur_r, patella_r	X Y Z
femur_l_length	LASI LMKNE, LGT LKNE	femur_l, patella_l	X Y Z
tibia_r_length	RKNE RANK, RMKNE RHEE	tibia_r	X Y Z
tibia_l_length	LNKE LANK, LMKNE LHEE	tibia_l	X Y Z
foot_horiz_l	LHEE LTOE	talus_l, calcn_l, toes_l	X Z
foor horiz_r	RHEE RTOE	talus_r, calcn_r, toes_r	X Z
foot_vertic_l	LHEE LANK, LHEE LMMA	talus_l, calcn_l, toes_l	Y
foot_vertic_r	RHEE RANK, RHEE RMMA	talus_r, calcn_r, toes_r	Y

Table C. Homologous points on the bone surfaces of the generic model and individual MRI. The whole set was initially identified on the generic model and saved in the global system of coordinates. Then the first group of landmarks were placed on the individual MRI and the second group were projected into the space of the MRI with the help of a Thin-Plate Spline transformation determined by the first landmark set. The second set were then checked and adjusted to fall onto predefined orientation planes through the body segments.

Count	Name	Body	Group	Count	Name	Body	Group
1	torso_origin_in_pelvis	pelvis	1	54	femur_r_anter_diaph_25	femur_r	2
2	ASIS_l	pelvis	1	55	femur_r_poster_diaph_25	femur_r	2
3	ASIS_r	pelvis	1	56	femur_r_anter_diaph_75	femur_r	2
4	PSIS_l	pelvis	1	57	femur_r_poster_diaph_75	femur_r	2
5	PSIS_r	pelvis	1	58	patella_r_in_femur_r	femur_r	2
6	pub_infer_c	pelvis	1	59	patella_lat_l	patella_l	2
7	pub_super_c	pelvis	1	60	patella_med_l	patella_l	2
8	ilium_l	pelvis	1	61	patella_l_anter	patella_l	2
9	ilium_r	pelvis	1	62	patella_sup_l	patella_l	2
10	femur_l_center	femur_l	1	63	patella_l	patella_l	2
11	knee_l_center	femur_l	1	64	patella_lat_r	patella_r	2
12	knee_l_lat	femur_l	1	65	patella_med_r	patella_r	2
13	knee_l_med	femur_l	1	66	patella_r_anter	patella_r	2
14	femur_r_center	femur_r	1	67	patella_sup_r	patella_r	2
15	knee_r_center	femur_r	1	68	patella_r	patella_r	2
16	knee_r_lat	femur_r	1	69	ankle_l_lat	tibia_l	2
17	knee_r_med	femur_r	1	70	ankle_l_med	tibia_l	2
18	fibula_l_lat_malleol_tip	tibia_l	1	71	ankle_l_center	tibia_l	2
19	tibia_l_med_malleol_tip	tibia_l	1	72	talus_l_center_in_tibia	tibia_l	2
20	fibula_r_lat_malleol_tip	tibia_r	1	73	tibia_l_center	tibia_l	2
21	tibia_r_med_malleol_tip	tibia_r	1	74	tibia_l_lat	tibia_l	2
22	femur_l_center_in_pelvis	pelvis	2	75	tibia_l_med	tibia_l	2
23	femur_r_center_in_pelvis	pelvis	2	76	tibia_l_epiph_yellow_anterior_plate	tibia_l	2
24	sacroiliac_l	pelvis	2	77	tibia_l_epiph_yellow_patel_tend_point	tibia_l	2
25	sacroiliac_r	pelvis	2	78	tibia_l_epiph_yellow_posteriormost	tibia_l	2
26	5th_sacr_v	pelvis	2	79	tibia_l_ankle_yellow_anter	tibia_l	2
27	AIIS_l	pelvis	2	80	tibia_l_epiph_green_medial_lower_epiph	tibia_l	2
28	AIIS_r	pelvis	2	81	fibula_l_as	tibia_l	2
29	isch_infer_l	pelvis	2	82	tibia_l_midshaft_anter	tibia_l	2
30	isch_infer_r	pelvis	2	83	tibia_l_midshaft_poster	tibia_l	2
31	isch_spine_l	pelvis	2	84	fibula_l_midshaft_anter	tibia_l	2
32	isch_spine_r	pelvis	2	85	tibia_l_anter_diaph_25	tibia_l	2
33	isch_tuber_l	pelvis	2	86	tibia_l_anter_diaph_75	tibia_l	2
34	isch_tuber_r	pelvis	2	87	fibula_l_anter_diaph_75	tibia_l	2
35	gr_troch_as_l	femur_l	2	88	ankle_r_lat	tibia_r	2
36	gr_troch_lat_l	femur_l	2	89	ankle_r_med	tibia_r	2
37	gr_troch_ps_l	femur_l	2	90	ankle_r_center	tibia_r	2
38	ls_troch_l	femur_l	2	91	talus_r_center_in_tibia	tibia_r	2
39	knee_l_center_in_femur_l	femur_l	2	92	tibia_r_center	tibia_r	2
40	femur_l_midshaft_anter	femur_l	2	93	tibia_r_lat	tibia_r	2
41	femur_l_midshaft_poster	femur_l	2	94	tibia_r_med	tibia_r	2
42	femur_l_anter_diaph_25	femur_l	2	95	tibia_r_epiph_yellow_anterior_plate	tibia_r	2
43	femur_l_poster_diaph_25	femur_l	2	96	tibia_r_epiph_yellow_patel_tend_point	tibia_r	2
44	femur_l_anter_diaph_75	femur_l	2	97	tibia_r_epiph_yellow_posteriormost	tibia_r	2
45	femur_l_poster_diaph_75	femur_l	2	98	tibia_r_ankle_yellow_anter	tibia_r	2
46	patella_l_in_femur_l	femur_l	2	99	tibia_r_epiph_green_medial_lower_epiph	tibia_r	2
47	gr_troch_as_r	femur_r	2	100	fibula_r_as	tibia_r	2
48	gr_troch_lat_r	femur_r	2	101	tibia_r_midshaft_anter	tibia_r	2
49	gr_troch_ps_r	femur_r	2	102	tibia_r_midshaft_poster	tibia_r	2
50	ls_troch_r	femur_r	2	103	fibula_r_midshaft_anter	tibia_r	2
51	knee_r_center_in_femur_r	femur_r	2	104	tibia_r_anter_diaph_25	tibia_r	2
52	femur_r_midshaft_anter	femur_r	2	105	tibia_r_anter_diaph_75	tibia_r	2
53	femur_r_midshaft_poster	femur_r	2	106	fibula_r_anter_diaph_75	tibia_r	2

Table D. Key Supporting Scripts and Utilities

File Name	Appears in Notebook(s)	Description
fibre_scale_script.py	4_update_generic_model_with_mri_data.ipynb	Fiber optimisation following Modenese et al. 2016
momentArms_plot.py	5_optimize_muscles.ipynb	Calculate and visualize muscle moment arms
optimization_grid.py	5_optimize_muscles.ipynb	Grid search for muscle via point optimization
rotation_utils.py	2.0_use_mri_data.ipynb, 2.1_after_controlling_mri_data.ipynb	Utils for rotation
simFunctions.py	3_scale_generic_model.ipynb, 4_update_generic_model_with_mri_data.ipynb	Utils for SIM functions
stan_utils.py	1_extract_static_C3D.ipynb, 3_scale_generic_model.ipynb	Utils for various functions
tps_scripts.py	2.0_use_mri_data.ipynb, 2.1_after_controlling_mri_data.ipynb	Functions for thin-plate spline warping
wrap_scripts.py	4_update_generic_model_with_mri_data.ipynb	Functions for wrapping surface collision detection and adjustment

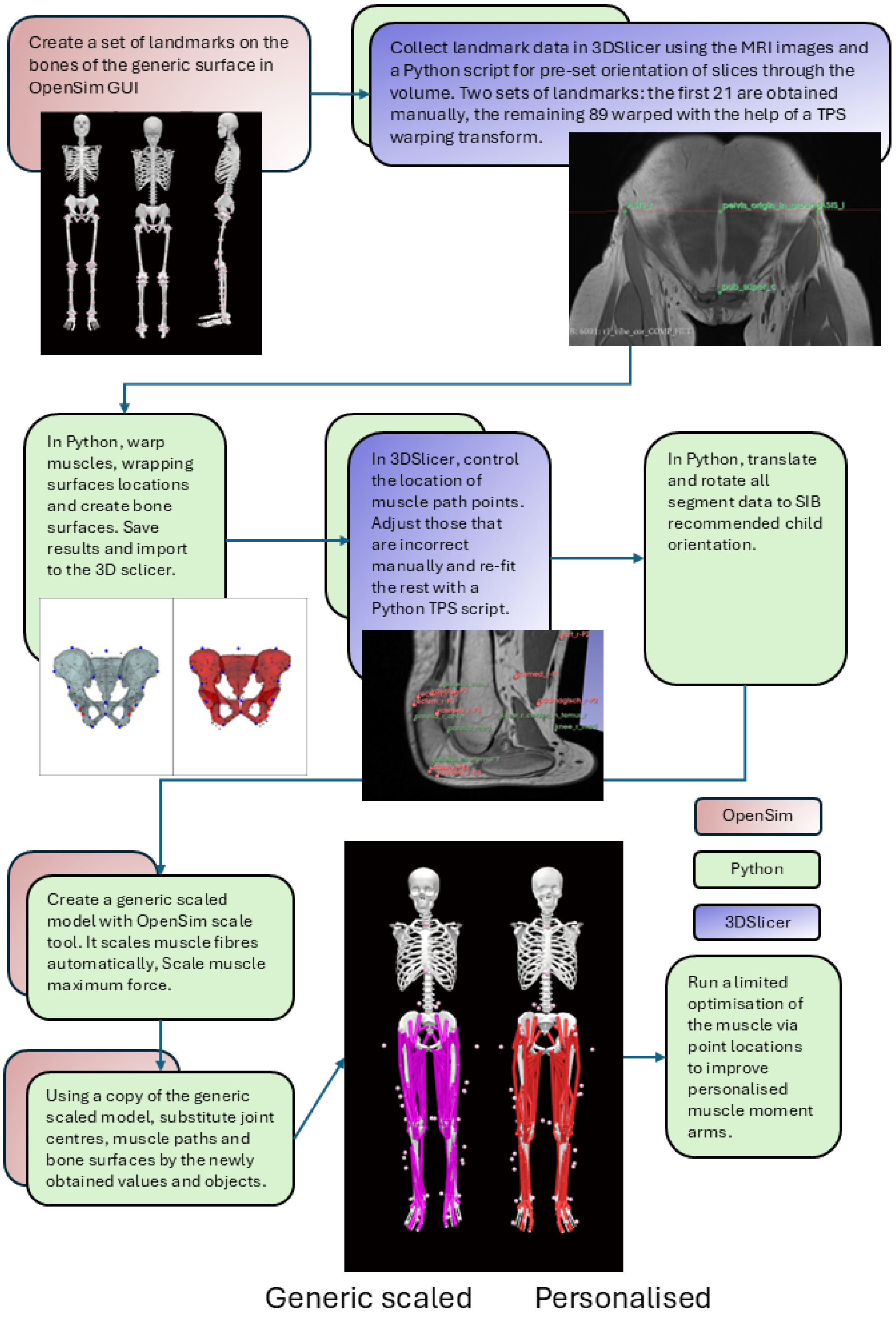

Figure B. The workflow for creating personalized models. Model views have been generated with the help of a generic model by Rajagopal et al. (Rajagopal A, Dembia CL, DeMers MS, Delp DD, Hicks JL, Delp SL. Full-Body Musculoskeletal Model for Muscle-Driven Simulation of Human Gait. IEEE Trans Biomed Eng. 2016;63: 2068–2079.) 3D mesh visualizations have been generated by ES using PyVista (open-source Python library, MIT License), PyVista license: https://github.com/pyvista/pyvista/blob/main/LICENSE; the MRI view of the pelvis and knee was created by ES with the help of 3D Slicer (Fedorov A, Beichel R, Kalpathy-Cramer J, Finet J, Fillion-Robin J-C, Pujol S, et al. 3D Slicer as an image computing platform for the Quantitative Imaging Network. Magn Reson Imaging. 2012;30: 1323–1341.)

3 MUSCLE PATHS AND JOINT REACTION FORCES COMPARISON

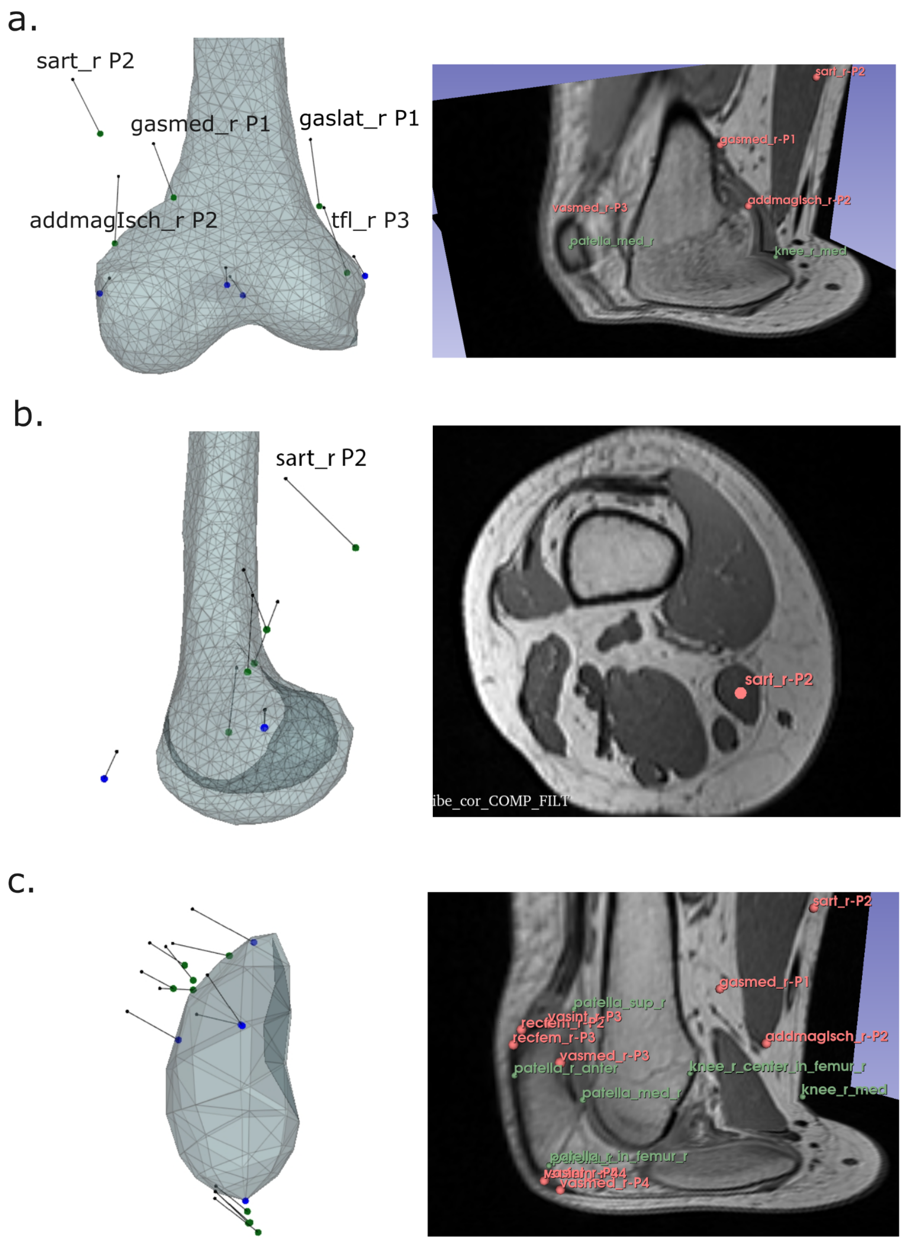

Figure C. Comparison of muscle path points between the generic and personalised models. The surfaces represent reconstructed bones from the personalised model. In the left-hand images, small black points indicate the muscle path points of the scaled generic model. These are connected by lines to corresponding coloured points on the personalised model—blue for bone landmarks and green for muscle paths. On the right, MRI snippets show the same muscle point locations for visual reference. 3D mesh visualizations have been generated by ES using PyVista (open-source Python library, MIT License), PyVista license: https://github.com/pyvista/pyvista/blob/main/LICENSE; the MRI view of the knee was created with the help of 3D Slicer (Fedorov A, Beichel R, Kalpathy-Cramer J, Finet J, Fillion-Robin J-C, Pujol S, et al. 3D Slicer as an image computing platform for the Quantitative Imaging Network. Magn Reson Imaging. 2012;30: 1323–1341.)

Table E. Muscles, whose moment arm differences between generic-scaled and MRI-based models exceeded 50% of the generic-scaled model moment arm range at one or more points across the joint angle range. Right side.

	**P01**	**P02**	**P05**	**P07**	**P20**
hip_flexion_r	glmax1_r, glmax3_r, glmin1_r, glmin2_r, glmin3_r, iliacus_r, piri_r	glmin1_r, glmin2_r, glmin3_r, piri_r	glmin3_r	glmin2_r, glmin3_r	glmin1_r, glmin2_r, glmin3_r, iliacus_r, piri_r, psoas_r
hip_adduction_r	glmax1_r, glmax2_r, glmed3_r, glmin3_r, psoas_r	addbrev_r, addlong_r, glmax1_r, glmax2_r, glmed2_r, glmin1_r, glmin2_r, glmin3_r, psoas_r	addlong_r, glmax1_r, glmax2_r, glmed2_r, glmin3_r	addlong_r, glmax1_r, glmax2_r, glmed2_r, glmin3_r, tfl_r	glmax1_r, glmax2_r, glmed1_r, glmed2_r, glmin2_r, glmin3_r, iliacus_r
hip_rotation_r	glmax3_r, glmed1_r, glmin2_r, psoas_r, semimem_r	addbrev_r, bflh_r, glmax3_r, psoas_r, semimem_r, semiten_r	glmax1_r, glmax3_r, glmed1_r, grac_r, semimem_r, semiten_r	bflh_r	addmagMid_r, addmagProx_r, bflh_r, psoas_r, sart_r, semimem_r
knee_angle_r	bflh_r, gasmed_r	bflh_r, gasmed_r		bfsh_r, gasmed_r, grac_r, semiten_r	gasmed_r, sart_r

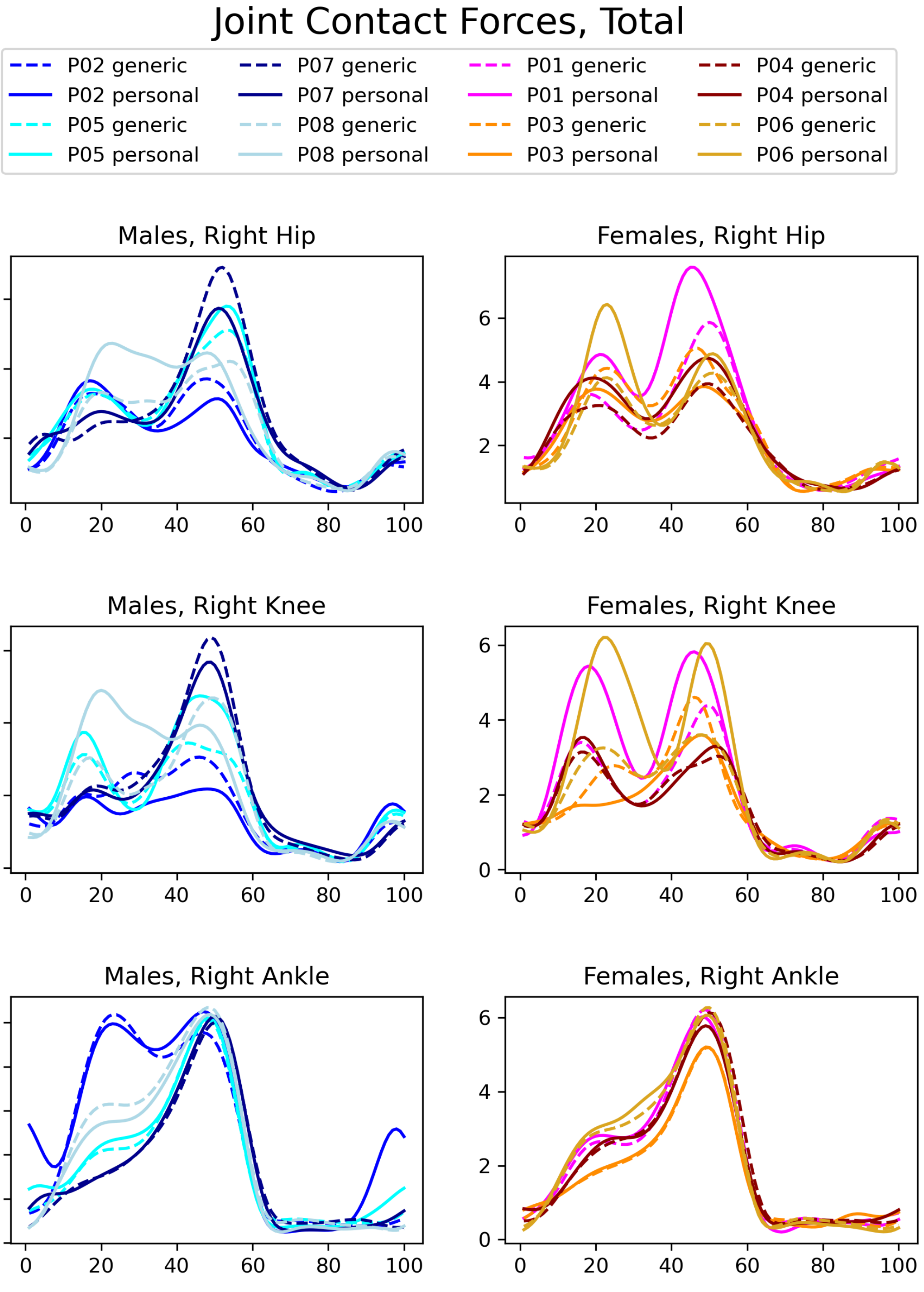

Figure D. Joint contact forces for generic-scaled and MRI-based models of each individual. Individuals are differentiated by colour Males are in the left and females in the right columns. Dashed lines signify generic-scaled and solid lines show MRI-based models.

4 MODEL VALIDATION

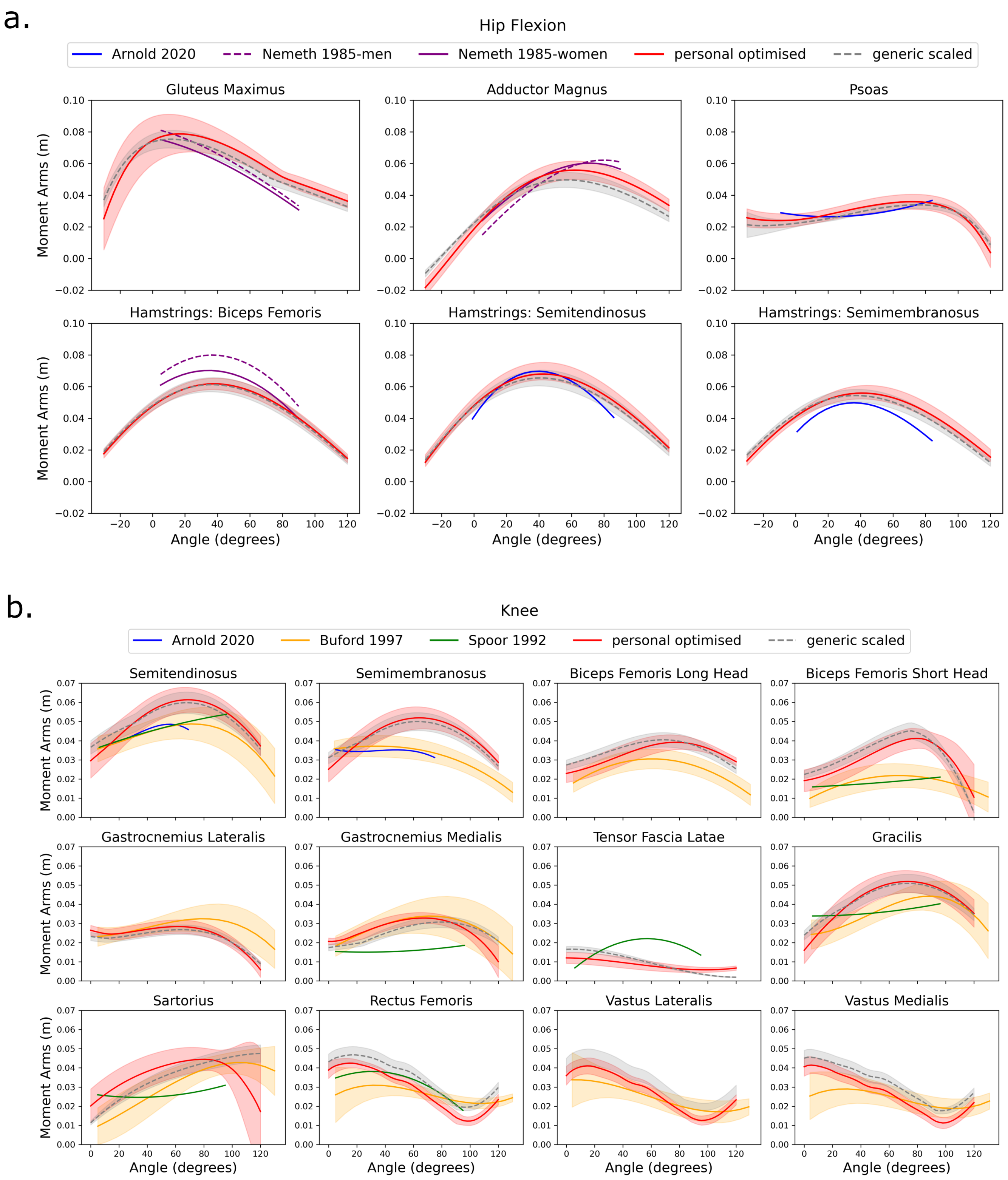

Figure E. Comparison of muscle moment arms with published cadaveric data.

5 MUSCLE ACTIVATIONS AND EMG DATA

Average muscle activations for the right foot gait cycle in MRI-based models and generic scaled across all individuals, alongside rectified and filtered experimental EMG data.

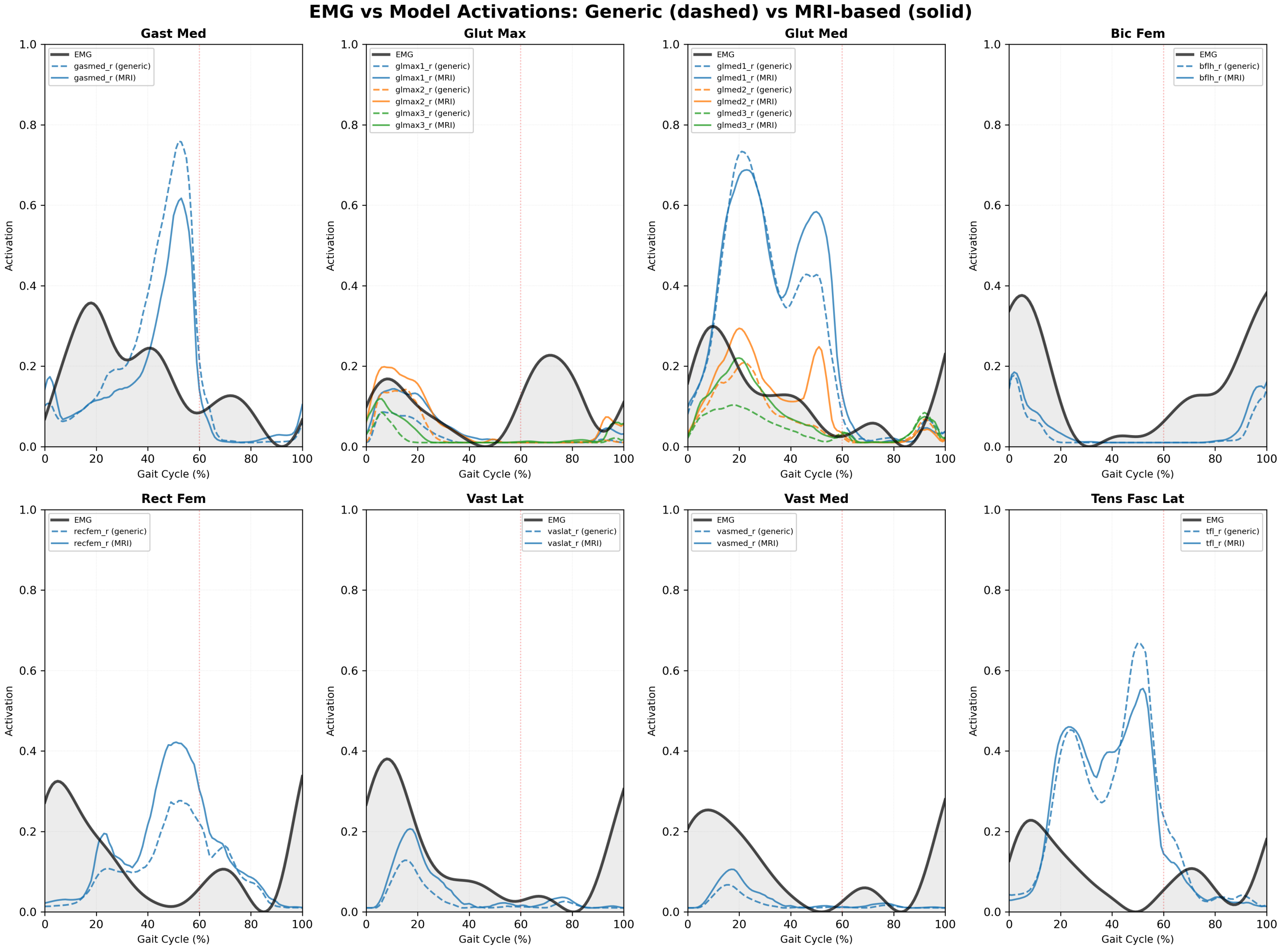

Figure F. Average muscle activations for a right foot walking cycle in a MRI-based and generic scaled models across individuals.
