# Supplementary material for: Do we need medical imaging-informed musculoskeletal models for simulations in healthy adults? A new workflow based on magnetic resonance imaging highlights the importance of personalized geometry
Source: PLoS Comput Biol. 2026 Mar 16;22(3):e1014073. doi: 10.1371/journal.pcbi.1014073 (PMC13029800; doi:10.1371/journal.pcbi.1014073)
Supplement: Attachment — Submitted filename: rebuttal_letter.pdf [file pcbi.1014073.s004.pdf]

To the Editors

PLOS Comutational Biology

26. September 2025

Rebuttal letter (initial submission PCOMPBIOL-D-25-01448)

Summary of previous feedback from the editor Emma Claire Robinson:

At this time we have decided to ***reject with option to resubmit****. Please clarify main contributions with view of a more general audience and resubmit the paper with a working link to the code. We will then be happy to take another look.*

Authors‘ comment:

Thank you for your valuable feedback. We have carefully addressed the concerns raised in your comments and made the necessary modifications to the manuscript (see the list of changes below).

Changes:

1) We have changed the title and re-written the abstract and whole manuscript to better highlight the scientific contributions of our paper, making it accessible to both the general scientific audience and the biomechanics community.

2) The link to download the developed workflow, including sample data, has been fixed and is now fully functional.

3) We corrected the typo you pointed out (e.g., "In articular" has been corrected to "In particular") and carefully reviewed the manuscript to eliminate other typographical errors.

4) We added detailed information about muscle wrapping surfaces to clarify this terminology and ensure the manuscript is more comprehensible to a broader audience.

5) The main contributions of the paper have been clarified and emphasized throughout the text to improve readability and better communicate the significance of our work.

Sincerely,
Ekaterina Stansfield, Willi Koller, Basílio Gonçalves, and Hans Kainz
