# Supplementary material for: Do we need medical imaging-informed musculoskeletal models for simulations in healthy adults? A new workflow based on magnetic resonance imaging highlights the importance of personalized geometry
Source: PLoS Comput Biol. 2026 Mar 16;22(3):e1014073. doi: 10.1371/journal.pcbi.1014073 (PMC13029800; doi:10.1371/journal.pcbi.1014073)
Supplement: Attachment — Submitted filename: ReviewerComments.docx [file pcbi.1014073.s002.docx]

The submitted manuscript describes and attempts to validate/verify the resulting models by comparing results from typical musculoskeletal modelling steps. I would like to start by congratulating the authors on the mammoth tasks of the work they have presented here – not only in the actual simulations but also in the documentation provided and sharing of the workflow. This is the kind of work needed to continue to elevate the field of biomechanics. I nevertheless have some comments and questions based on the submitted manuscript which I believe warrant some form of discussion or at minimum a clarification. I have listed my “major” comments in general comments sections and smaller grammar or inconsistencies in the “specific comments”. I look forward to receiving an updated manuscript and the responses to my questions.

General comments;

Without a comparison of the bone geometry – even in terms of some specific geometrical features such a length, femoral torsion angle for example I fear that a naïve audience may consider the current implementation an exercise showing that if you modify your model- and results will change - or at minimum a discussion as to why it is not relevant.

Reporting of geometrical differences:

Results Line 126 🡪 Perhaps it is my naivety but I am curious as other readers may be, why principal component analysis is the most appropriate method for these comparisons .

Figure 1: I appreciate the effort the authors have made in trying to describe the differences between the two modelling approaches, and I do not doubt the large effort and thought put into these figures – however if I read your paper – and the title I would rather expect the differences to be presented/calculated on a subject-subject basis and having this difference shown in such figures.

My knee jerk reaction is that there is going to be high(er) similarity between the linear scaled models –and the variability in the MRI-based models will be much larger. So instead of representing it as PCs (taking away the personalization element which is the core of your paper) – could you use the approach to quantify the difference between scaled and personalised models. I am happy to be convinced otherwise however at this point it remains quiet hard to read on a personalised level.

Suppl material

I would like to commend the authors on the efforts in reporting as completely as	possible the different simulation results. The one additional comparison which I would like to see – just to confirm that the reason you see the differences which you report in contact forces are the muscle activations in those muscles where you have the EMG recordings. These are listed in the manuscript in Appendix but were not present in the supp material.

Specific comments:
Abstract

line 27-29: the detail of “to map 106 homologous landmarks from generic models onto participants anatomy “ seems to specific for the abstract. It gives a very specific methodology but does not fully explain how this approach was beneficial in actually building the models. Consider removing

Line 31-32: “ were compared using principal component analysis, joint kinematics, and joint contact force analyses.” Consider stating the variables you compare rather than the methods you used to generate them.

Introduction:

Lines 101-111: This paragraph seems a little too focus on the methods you will later utilise – in the methods section – I would suggest restriction this section to the explanation of previous approaches and their short comings. Some parts may be moved to the later paragraph on line 113🡪

Methods

line 371: Did you do or consider any inter-operator reproduceability – or at the least how an error in identifying these points can propagate through you model development ?

Line 442 “predicted joint contact forces” – consider using the term “estimated” or “calculated” instead of predicted as your models are physics based to differentiate it from any naïve interpretation this was a “AI” prediction.

Line 444 “personalisation.” – from my reading you used “personalized” – please check for consistency – also with optimisation and optimization

Line 445: Consider renaming this “Verifcation” instead of “validation” as some of your parameters you do not a TRUE gold standard

Did you perform any adjustments to your marker model on the two models – in particular the MRI model after personalisation – I suspect this will drive the majority of your kinematics differences rather than anything to do with the anatomy

Results

Line 160: sartorius not italicised

Clarification: The mass/inertial properties of the personalised model were not altered – meaning any change in your dynamics is due only to the change in kinematics – is this true ?

Line 192 – 200: What is the added value of going back to the PC’s here ? Figure 3 is already, at least for me, enough to confirm the differences between the two models.

Line 204: validation vs verification

Something missing from your results section is a short explanation of the processing times for building your personalised models and what type of computing was used.
