# Supplementary material for: Do we need medical imaging-informed musculoskeletal models for simulations in healthy adults? A new workflow based on magnetic resonance imaging highlights the importance of personalized geometry
Source: PLoS Comput Biol. 2026 Mar 16;22(3):e1014073. doi: 10.1371/journal.pcbi.1014073 (PMC13029800; doi:10.1371/journal.pcbi.1014073)
Supplement: Attachment — Submitted filename: Composite_Reviewer_Comments_with_Answers.docx [file pcbi.1014073.s005.docx]

LINE REFERENCES IN THE REVIEWER ANSWERS:

Reviewer #1:

The submitted manuscript describes and attempts to validate/verify the resulting models by comparing results from typical musculoskeletal modelling steps. I would like to start by congratulating the authors on the mammoth tasks of the work they have presented here – not only in the actual simulations but also in the documentation provided and sharing of the workflow. This is the kind of work needed to continue to elevate the field of biomechanics. I nevertheless have some comments and questions based on the submitted manuscript which I believe warrant some form of discussion or at minimum a clarification. I have listed my “major” comments in general comments sections and smaller grammar or inconsistencies in the “specific comments”. I look forward to receiving an updated manuscript and the responses to my questions.

General comments;

Without a comparison of the bone geometry – even in terms of some specific geometrical features such a length, femoral torsion angle for example I fear that a naïve audience may consider the current implementation an exercise showing that if you modify your model- and results will change - or at minimum a discussion as to why it is not relevant.

*Thank you for your positive assessment and thoughtful engagement with our manuscript.*

*To answer your main concern, we have re-worked Figure 1 and the text of the results to include a more extensive comparison of the bone geometry (L.158-179). The PC analysis, which is also a form of comparison of bone geometry that determines main trends in variation as opposed to individual differences, has been given a separate space. Implications of its results are discussed in the text and repeated below (L. 209-226).*

*We answer your further questions in the order of their appearance.*

Reporting of geometrical differences:

Results Line 126 🡪 Perhaps it is my naivety but I am curious as other readers may be, why principal component analysis is the most appropriate method for these comparisons.

*Principal component analysis has been an accepted and essential step in comparative morphology, where data is represented by either 2D or 3D anatomical landmarks. Large summary works include Slice* [*(2005)*](https://paperpile.com/c/rhk06q/vwTEl/?noauthor=1) *and Zeldich et al.* [*(2012)*](https://paperpile.com/c/rhk06q/5sEuN/?noauthor=1)*. We used this approach to highlight the general trend of the differences between models. Here, PC1 summarised the largest percent of variation in the sample and differentiated between Generic-scaled and MRI-based models. In other words, the difference between models of the same individual was found to be larger than the difference between two models of the same individual (L. 209-226).*

*We have now separated linear measurement from the PCA of bone landmarks and muscle paths, and shifted PCA figure to the second position (Figure 2).*

Figure 1: I appreciate the effort the authors have made in trying to describe the differences between the two modelling approaches, and I do not doubt the large effort and thought put into these figures – however if I read your paper – and the title I would rather expect the differences to be presented/calculated on a subject-subject basis and having this difference shown in such figures.

My knee jerk reaction is that there is going to be high(er) similarity between the linear scaled models –and the variability in the MRI-based models will be much larger. So instead of representing it as PCs (taking away the personalization element which is the core of your paper) – could you use the approach to quantify the difference between scaled and personalised models. I am happy to be convinced otherwise however at this point it remains quiet hard to read on a personalised level.

*We present a new version of Figure 1 with person-by-person differences between the Generic-scaled and MRI-based models. Please note that the linear and angular differences have been obtained by subtracting Generic-scaled from MRI-based measurements. In contrast, differences of landmark (or point) positions in 3D are reported as the magnitude of the vector between the two points (i.e., Euclidean distance). Panels a and c show plots of lengths and widths of skeletal segments and femur and tibia angles. Panels b and d show differences between muscle points (origins, attachments, and via-points) and joint locations. We also supply a model-by-model comparison of joint and muscle via-point locations in the ground frame, as well as plots of normalised moment arms over one gait cycle for a selected set of muscles in the Supplementary Information, Figure S1.*

Suppl material

I would like to commend the authors on the efforts in reporting as completely as	possible the different simulation results. The one additional comparison which I would like to see – just to confirm that the reason you see the differences which you report in contact forces are the muscle activations in those muscles where you have the EMG recordings. These are listed in the manuscript in Appendix but were not present in the supp material.

*Thank you for your positive remark. In response to your question, we have now combined all Supplementary Information in one document, where EMG signals are plotted against activations of the respective muscles for each individual in the’* *Muscle Activations and EMG Data’ subsection (Figure S6).*

Specific comments:
Abstract

line 27-29: the detail of “to map 106 homologous landmarks from generic models onto participants' anatomy “ seems to specific for the abstract. It gives a very specific methodology but does not fully explain how this approach was beneficial in actually building the models. Consider removing.

*We have simplified the said sentence while keeping the cornerstone information about the specific method present (L. 26-30).*

*“Our workflow concentrates on creating subject-specific joint centers and muscle paths. It also reconstructs bone surfaces without requiring MRI segmentation. It integrates 3D Slicer and Python scripts, and uses Thin-Plate Spline (‘TPS’) mapping of anatomically equivalent (‘homologous’) landmarks from generic models onto participants' anatomy. ”*

Line 31-32: “ were compared using principal component analysis, joint kinematics, and joint contact force analyses.” Consider stating the variables you compare rather than the methods you used to generate them.

*The sentence has been changed to “Simulations were performed using participants' 3D motion capture data, and the two model types were compared using their geometry, joint kinematics and dynamics during one walking cycle, and resultant joint contact forces” (Lines 31-34).*

Introduction:

Lines 101-111: This paragraph seems a little too focus on the methods you will later utilise – in the methods section – I would suggest restriction this section to the explanation of previous approaches and their shortcomings. Some parts may be moved to the later paragraph on line 113🡪

*We have expanded this part of the Introduction, adding more detail on related methods for personalization of musculoskeletal model morphology. In contrast, we reduced the amount of information on our methodology, restricting it to the major points of difference with the published methods (L. 110-145).*

Methods

line 371: Did you do or consider any inter-operator reproduceability – or at the least how an error in identifying these points can propagate through you model development?

*This is a very good point. Data for the presented manuscript have been collected by one individual. Several students and co-authors have tried and tested the approach. However, we do not presently have a comparative set of data with different researchers creating models for the same participant. We added text explaining the current situation (L. 548-552):*

*“Assessing the reliability of the whole workflow was beyond the scope of this study and should be done in future investigations. However, we qualified the intra-observer reliability for locating bony landmarks. The mean difference across all bone landmarks for all individuals was 2.06 ± 1.86 mm. The mean difference across patella landmarks was 1.06 ± 0.37 mm.”*

Line 442 “predicted joint contact forces” – consider using the term “estimated” or “calculated” instead of predicted as your models are physics-based to differentiate it from any naïve interpretation this was a “AI” prediction.

*Changed.*

Line 444 “personalisation.” – from my reading you used “personalized” – please check for consistency – also with optimisation and optimization.

*Changed.*

Line 445: Consider renaming this “Verifcation” instead of “validation” as some of your parameters you do not a TRUE gold standard.

*Changed.*

Did you perform any adjustments to your marker model on the two models – in particular the MRI model after personalisation – I suspect this will drive the majority of your kinematics differences rather than anything to do with the anatomy.

*The skin markers were initially warped onto the MRI-based model with the help of the same TPS transformation as was used for the muscle paths. These were then utilised as a template for the Inverse Kinematics fitting of the experimental markers with the help of the OpenSim scaling tool, where the scaling option was set to ‘False’ and marker mapping to ‘True’. No further adjustments were made to the skin markers.*

*We added this information in the MRI-based model subsection of the Methods section (L.518-522) :*

*“The skin markers were initially warped onto the MRI-based model using the same TPS transformation as was used for the muscle paths. These were then utilised as a template for the Inverse Kinematics fitting of the experimental markers with the help of the OpenSim scaling tool, where the scaling option was set to ‘False’ and marker mapping to ‘True’. No further adjustments were made to the skin markers.”*

Results

Line 160: sartorius not italicised

*Changed.*

Clarification: The mass/inertial properties of the personalised model were not altered – meaning any change in your dynamics is due only to the change in kinematics – is this true?

*Correct, mass and inertial properties were kept the same as in the Generic-scaled model. This was a deliberate choice on our part that only the geometry of the model changes between the Generalised-scaled and MRI-based models to enable a clear understanding of the effect of geometry, i.e. muscle moment arms, only.*

*We added a clarification of this point in the manuscript (L.543-547):*

*“We chose to keep musculotendon parameters (tendon slack length and optimal fiber length) and segment inertial properties in the MRI-based models the same as in the generic-scaled models to ensure that any observed differences between the two model types were due to geometry only. Initially, we did not expect to obtain noticeable differences in limb segment lengths between the two model types.”*

Line 192 – 200: What is the added value of going back to the PC’s here ? Figure 3 is already, at least for me, enough to confirm the differences between the two models.

*This analysis shows that the models differ not only in geometry but also in their functional outputs. The principal component 1 (PC1) differentiates the shapes of generic-scaled and MRI-based models. This trend correlates with joint contact forces so that generic-scaled models systematically produce lower values.*

Line 204: validation vs verification

*Changed in line 277.*

Something missing from your results section is a short explanation of the processing times for building your personalised models and what type of computing was used.

*Thank you for this comment. The processing times are mostly dependent on the operator’s knowledge of the anatomy for the correct bone landmark placement, and then for the control of the position of the muscle path points.*

*We added the following text on Lines 428-434: “ The complete pipeline with all scripts, detailed documentation, and a worked example is publicly available at https://github.com/katya-stanzy/thin-plate-spline_personalised_muscoloskeletal_model. Workflow steps 1-9 correspond to the pipeline flowchart in Fig. S1 and the repository flowchart.png. Complete instructions, environment files, and example data are provided in the repository to facilitate reproduction. An experienced operator requires approximately 2 hours to create an MRI-based model using this pipeline.”*

******************************************************************************************

Reviewer #2: GENERAL COMMENT

This paper presents on a novel pipeline to enable the faster generation of personalized musculoskeletal models from medical imaging and motion capture data, exploiting

various software and without requiring images to be segmented beforehand. The entire pipeline was tested on data from 8 healthy adults, and a working example is

provided at the project page on GitHub (link available in text).

This topic is of particular interest to the biomechanics community, as demonstrated by the number of workflows and pipelines developed over the last few years to develop

musculoskeletal models with different degrees of personalization. Indeed, generic scaled models are typically chosen to minimize the efforts, both in terms of cost and

time required for data acquisition and processing, but fail to capture many features that can make a difference on the simulations. This is more apparent for subjects with

musculoskeletal or neuromuscular disorders but holds true for healthy individuals.

The rationale for this study and for the development of a new pipeline to generate personalized musculoskeletal models is clear and well laid out, and it is understandable.

The reported results are encouraging. However, the introductory section could be improved and expanded to include some more references to current/existing pipelines to develop musculoskeletal models, possibly highlighting their main limitations. Furthermore, the methods section lacks the level of detail required to ensure a full understanding and to replicate the procedure. Consequently, also the discussion section should be revised, as I believe that some key points are not sufficiently discussed.

*Thank you very much for reviewing our manuscript and supporting it with detailed comments. We have expanded the introduction section with further references and their descriptions (L.110-130).*

Please see specific comments below.

SPECIFIC COMMENTS

- Abstract -

Please consider adding some numbers to quantify the observed differences, even in the abstract.

*Summaries for geometric dimensions, kinematics, and knee joint contact forces have been added to the abstract and to the results (L. 35-47 and 158-170).*

- Introduction -

The introduction is good in general but could be improved. One critical aspect that is not touched on is how could one justify the need for acquiring MRI data on healthy participants, considering the costs and the organizational issues that may be connected to it.

*This is an important point – thank you for calling it out. Overall, the choice to supplement musculoskeletal modeling with medical imaging depends on the research aim and question. Personalized models may not be needed for general kinematics-driven analyses, e.g., comparing forces between walking and running. However, imaging-based personalization becomes necessary when addressing anatomy-specific questions, e.g., how femoral anteversion differs between sexes and how this affects joint forces. Our paper highlights uncertainties that should be considered when using generic models. Our approach has practical advantages: many clinical datasets already include both 3DGA and MRI data depending on the condition under study, yet these imaging data are typically not used for model creation. Hence, the required data may already be available. Additionally, MRI's lack of ionizing radiation makes it a safer alternative to imaging modalities such as CT.*

*We added these points to the Discussion (L.371-376 and 383-388).*

>Lines 97-100: Additionally, models' muscle fiber parameters [...] for routine use.

Comment: Please be more specific, for the readership to know. Consider adding some examples of data and parameters that can be extracted from medical imaging data.

Also, it should be clearly said that some of these methods cannot be used for all muscles (e.g., ultrasound imaging for deep muscles).

*We added information on the role of ultrasound and dynamic ultrasound in determining muscle fiber parameters (L. 105-109).*

Comment: To better stress this point (i.e., that methods for personalization are typically time-consuming and manual), provide some more details to the readers. For

example, how much time is required roughly to collect and process medical imaging data, or for other pipelines to generate a musculoskeletal model?

*Papers do not usually report the time it takes to create this model. However, the last author of the present manuscript has extensive experience in creating MRI models with different pipelines. It usually depends on the image quality and experience of the researcher. To create their first MRI model, the last author needed approximately three weeks. With more experience, most workflows still take several days.*

*A comment was added in Methods, L.433-434:*

*“An experienced operator requires approximately 2 hours to create an MRI-based model using this pipeline.”*

>Lines 101-102: [...] such as 3D Slicer for segmentation and annotation and OpenSim for musculoskeletal modeling [...].

Comment: Please add references for both 3D Slicer and OpenSim, and to works using these tools to develop (personalized) musculoskeletal models – if any.

*The references were added (L.131-132).*

>Lines 109-111: [...] but our approach as 3D Slicer for segmentation and annotation and OpenSim for musculoskeletal modeling [...].

Comment: This paragraph does not add much to the introduction and repeats what came before. Consider removing it. Also, not many readers may be familiar with the

Thin-Plate Spline transformation: please add some references. Please be careful in using the term 'fully personalised'.

*Thank you for this comment. We would like to clarify that the referred sentence points to the key difference between SSM and the proposed method: instead of segmenting bone surfaces, we collect information on a sparse number of key anatomically homologous points on the bones. This proves to be sufficient for recreating bone shape following geometric morphometrics approaches. We have now simplified the language of the paragraph and added two references to the related method* [*(Bookstein 1991; Boyd et al. 1999)*](https://paperpile.com/c/rhk06q/72cxe+UpzPb)*.*

*L. 141-145: “*This landmark-based approach enables non-affine 3D transformation [[30,32]](https://paperpile.com/c/09IS7w/e11P+4bbQ) that maps both bone geometry and muscle-tendon paths from generic models onto individual anatomy, creating models that are personalized in both skeletal and muscular morphology while remaining accessible to researchers without extensive computational resources or reference databases.*”*

- Results -

>Lines 121-122: Each of the eight participants (four males and four females, healthy, from 26 to 51 years of age) [...].

Comment: Provide more details on the tested population. The information in Table 1 can be easily included here in brackets.

*This information was added.*

>Line 128-129: Bony landmarks and muscle paths of generic-scaled and MRI-based models were analysed using principal components.

Comment: Please provide the rationale for combining both anatomical and muscle points in one single PCA.

*We added a sentence to explain the reason for combining the two sets of points in Methods (L. 579-585): “ The combination of bone and muscle path points in one analysis is justified by their intrinsic anatomical connection, which was maintained through the non-affine TPS transformation.”*

>Lines 140-143

Comment: Not all these features can be easily appreciated on the figure. Would it be possible to add some zoomed images to highlight lines between points or black dots?

*We have re-worked the display of PCA results (Figure 2) to include PC1 and PC2 with a larger format. We also added a text description of the meaning captured by the first two PCs in the text (L. 209-221):*

“*The first two principal components (PCs) described 63.9% of the variance (Fig. 2a). PC1 (51.0% of variance) strongly differentiated between MRI-based and generic-scaled models with no overlap between the two groups. For the pelvis, this component differentiated between shapes with a narrow distance between the ischial bones and a relatively posterior position of the torso origin, as in the generic-scaled models, from those with a relatively wider distance between ischiopubic arches, wider distance between hip joint centers and a relatively anterior position of the torso origin, as in the MRI-based models (Fig. 2b). For the limb segments, this component differentiated models with a relatively shorter femur and a longer tibia, as in the generic-scaled models, from those with a relatively longer femur and a shorter tibia, as in the MRI-based models (Fig. 2b). PC2 (12.0% of variance) differentiated MRI-based male and female models, where females tended to have a wider pelvis and a relatively shorted femur than males in our study cohort (Fig. 2c). Generic-scaled models did not differentiate between males and females.*”

>Lines 149-151: In most cases, [...] (Fig. 1c).

Comment: Is this to be attributed to the generic geometries in themselves, to a misplacement of the markers on the generic unscaled model and/or on the subject in the

gait lab, or to both? Please comment.

*The differences are due to the intrinsic differences in geometries. Given that principal components are ranked by the amount of variation they describe, the first two summarise the general trends in the data. Errors due to the misplacement of the markers are usually random and therefore not represented by these components. We added an appropriate clarification in the Results L. 222-225:*

*“Principal component analysis highlighted that the differences between the two model types for the same individual were larger than the differences between individuals. This is evident from the percentage of variance (51.0%) described by the first principal component, as opposed to PC2 (12.0% of variance), which captured differences between individuals”.*

>Lines 156-160.

Comment: How critical is it that muscle via points on generic scaled models are located further away from the knee joint centre? Does it have to do with different

underlying geometries and/or the absence of wrapping surfaces?

*MRI-based models had the same combination of wrapping surfaces as the generic-scaled. We did not include the analysis of wrapping surface radii in the Results and Discussion, as they were mostly inherited from the generic scaling. However, muscle via points were adjusted and, therefore, could interact with the wrapping surfaces in motion. We ensured that this does not happen by controlling for the wrapping surface radius (step 8, Table 2, L 436) and for the position of the nearest muscle via points and attachments (step 9, Table 2, L 436).*

*Indeed, the larger distance of some via points from the knee centre of rotation in the generic-scaled models may be responsible for the lower muscle forces required to create a sufficient moment to match given kinematics. However, the detailed analysis of which via point is vital at what time in which muscle was beyond the scope of the presented paper. We added an appropriate sentence to the Discussion L.307-316):*

*“Patellar size was another notable discrepancy in geometry between generic-scaled and MRI-based models. The patella in the Rajagopal model is relatively large; consequently, when scaled linearly with femoral dimensions, it resulted in larger patellar dimensions in the generic-scaled models than in the MRI-based models for some individuals.*

*Consequently, most muscle paths in generic-scaled models deviated from corresponding muscle locations visible in MRIs. These differences influenced muscle moment arms, with such muscles as tensor fascia latae, sartorius, gluteus maximus medial part, adductor magnus distal part, and psoas (mean difference 4.0*$\pm$*7.0 mm) with the largest discrepancies around the hip joint. Around the knee, sartorius, vasti, rectus femoris, and gastrocnemius medialis showed the largest deviations.”*

>Lines 163-168

Comment: How easily can one identify the landmarks on the patella? This could be a possible explanation for the large reported errors.

*Identification of patella landmarks has been standardised in our pipeline. The differences between generic-scaled and MRI-based models were mostly due to the different relative size of the patellas in study participants, when compared with the generic model. We added the following lines in Methods:*

*L. 548-552: “Assessing the reliability of the whole workflow was beyond the scope of this study and should be done in future investigations. However, we qualified the intra-observer reliability for locating bony landmarks. The mean difference across all bone landmarks for all individuals was 2.06 ± 1.86 mm. The mean difference across patella landmarks was 1.06 ± 0.37 mm.”*

>Lines 172-173: Knee and subtalar angles exhibited the least difference between models, and pelvis tilt was affected by the personalisation the most.

Comment: Please be more specific and quantify such differences. It is normal for some pelvis angles to be 'off' in MRI-based models, considering the supine position of

the subjects in the scanner. Although it should be later corrected.

*Having removed the cosine comparison between stride vectors (at the request of the first reviewer), we no longer have the ground for discussing pelvis coordinates. We have, therefore, removed the discussion of the pelvis tilt from the text.*

>Figure 2

Comment: Please report also the pelvis list, tilt and rotation angles.

*Following the suggestion, Figure 3 now provides pelvis coordinates as well as limb coordinates.*

>Lines 208-209

Comment: Is it any different for the generic-scaled models? Please specify.

*New plots of EMG and muscle activations averaged across individuals have been added in the Supplementary Information (Fig. S6). Appropriate information has been added in the manuscript, L. 285-287:*

*“In most cases, EMG activity corresponded to muscle activations obtained from the generic-scaled and MRI-based models (see Supplementary Information, Fig. S6), with activation patterns differing in magnitude but not in timing across the gait cycle.”*

>Line 212-213: Despite [...] muscle moment arms [...] similar to generic-scaled ones.

Comment: Similar how? In profile, values, both?

*Added ‘in the profile and magnitude‘, L. 282-284:*

“*Despite discrepancies in leg length and muscle path point locations, muscle moment arms in the MRI-based and generic-scaled models showed similar consistentcy with with literature data in both profile and magnitude (Supplementary information Fig. S5).”*

- Methods -

In general, the authors well described the pipeline and steps required for model personalization, although some additional details should be provided. For example, there

is no mention of the time required to collect and process the experimental data nor to generate the models using the proposed pipeline. This is quite relevant. In addition,

the authors do not explain if and how muscle parameters (i.e., tendon slack length and optimal fiber length) were updated in the MRI-based models, which is critical.

*Thank you for this comment. We have added information on the time required for model creation in Methods:*

*L. 433-434: “An experienced operator requires approximately 2 hours to create an MRI-based model using this pipeline.”*

*L. 543-547: “We chose to keep musculotendon parameters (tendon slack length and optimal fiber length) and segment inertial properties in the MRI-based models the same as in the generic-scaled models to ensure that any observed differences between the two model types were due to geometry only. Initially, we did not expect to obtain noticeable differences in limb segment lengths between the two model types.”*

L. 363-370: *“Our study has several limitations. We did not account for individual motor control strategies; EMG-informed approaches may reduce excessive muscle activations* [*[50]*](https://paperpile.com/c/09IS7w/Nv8Kf)*. Morphological changes in personalized models may require corresponding musculotendon parameter adjustments* [*[51,52]*](https://paperpile.com/c/09IS7w/ob8Wm+fKh2Y)*. We did not modify segment inertial properties when creating MRI-based models, though we expect negligible impact. While we lack ground truth data for our participants, instrumented prosthesis studies* [*[11]*](https://paperpile.com/c/09IS7w/Ud5qF) *suggest personalized models produce more accurate results. Despite these limitations, our findings demonstrate that personalization affects biomechanical outcomes even in healthy populations.”*

>Table 1

Comment: Please report individual subjects' information in here. Mean and standard deviation can be directly added in text (as suggested in other comments).

*This has been done.*

>Line 327: Gait data were collected [...].

Comment: Please be more specific. What kind of task were the subjects asked to perform (i.e., at which speed?) and how many trials were collected?

*This information has been added*.

*L. 414-514: “Participants walked across the lab stepping on three force plates at a self-selected speed. Seven trials with successful marker detection were selected for analysis”.*

>Lines 335-338.

Comment: Please add the name of the scanner manufacturer.

*This information has been added, L 418-419: “Siemens MAGNETOM Vida”*

>Line 358: = 47 × m × h + 1285

Comment: While the use of this formula makes perfect sense for the scaled model, I am not sure it provides a good approximation of the generic volumes on the unscaled

model. I would have computed the generic model's volumes by inverting the formula of the maximal isometric force, as all parameters are known. Please comment.

*We appreciate this thoughtful question. Rajagopal et al. (2016) use the regression equation from Handsfield et al. (2014) to estimate total lower limb volume, which they then partition into individual muscle volumes by multiplying by muscle-specific proportions (*$\phi^{m}$*). This means the generic model volumes are already based on the Handsfield regression, just applied indirectly.*

*For our volume ratio calculations, using the Handsfield equation directly is therefore consistent with the original model formulation. Importantly, because the muscle proportion (*$\phi^{m}$*) is identical for both generic and scaled models (defined in the Rajagopal model), it cancels out in the ratio calculation:*

$V_{scaled}/V_{generic}= {(V}_{lowe limb scaled}\times\phi^{m} )/ (V_{lower limb generic}\times\phi^{m} ) =V_{lowe limb scaled}/V_{lower limb generic}$

*Thus, our approach correctly captures the geometric differences in total lower-limb volume between models without requiring muscle-specific proportions.*

>Lines 360-362: The scaled-generic model, together with muscle parameters and inertia values, became the template for creating the MRI-based models for each

participant.

Comment: It seems the generic inertial parameters were used as template for the personalised musculoskeletal model, which can be considered a limitation (especially if

the generic scaled geometries differ from the real bony geometries). Please comment and provide a rationale for this choice. The inertial parameters likely affect the

system's dynamics.

*This has now been explained in the Methods (L 545-549) and discussed in the Discussion (L. 366-368).*

*L. 366-368: “We did not modify segment inertial properties when creating MRI-based models, though we expect negligible impact.”*

*L.* *543-547: “We chose to keep musculotendon parameters (tendon slack length and optimal fiber length) and segment inertial properties in the MRI-based models the same as in the generic-scaled models to ensure that any observed differences between the two model types were due to geometry only. Initially, we did not expect to obtain noticeable differences in limb segment lengths between the two model types.”*

>Lines 371-376: The primary group included 21 landmarks easily identifiable on MRI and essential for further steps, placed manually on MRI using 3DSlicer (Fig. 5).

Remaining landmarks were projected into MRI space using a Thin-Plate Spline (TPS) non-linear registration algorithm available in 3DSlicer. An operator verified the

correct positioning of registered landmarks to ensure alignment with predefined orientation planes within the MRI volume.

Comment: These steps appear to be very much operator-dependent in terms of final outcome. Have the authors performed an analysis to quantify this? Furthermore, I

think it is quite important to provide the time expenditure to complete the manual identification of the 21 landmarks.

*We have provided the time estimate in the text. Indeed, the result depends on the operator. However, as the process is standardised, the errors are expected to be minimal. We added the following lines in Methods:*

*L. 548-552: “Assessing the reliability of the whole workflow was beyond the scope of this study and should be done in future investigations. However, we qualified the intra-observer reliability for locating bony landmarks. The mean difference across all bone landmarks for all individuals was 2.06 ± 1.86 mm. The mean difference across patella landmarks was 1.06 ± 0.37 mm.”*

>Line 378

Comment: Please provide a rationale for the simultaneous use of via points and wrapping surfaces.

*We added the requested information to our manuscript.*

>Lines 382-384

Comment: Please ensure numbers are consistent. Earlier, in text, it was mentioned that 106 points were identified (now 21+89 = 110).

*Thank you for noticing this. An explanation has been added.*

>Lines 389-390: Only a few manual adjustments were necessary (Fig. 6).

Comment: Please be more specific, as Figure 6 does not seem to show the manual adjustments.

*Thanks for pointing that out. The exact list of points to be checked is given in the notebook ‘example/mri/control/control.ipynb’ for the user to follow in the process of working with the data. We added an explanation in Table 2, step 6*, and in L. 491-495:

*“These changes were needed due to muscle origin and insertion points sometimes ending inside the bone and via path points falling outside of the muscle body center. The full list of the muscle adjustments is given in the Python notebook ‘folder/example/mri/control/control.ipynb’ in the project repository”*

>Line 403: During adjustment, a custom Python script [...].

Comment: This script is not available online, but I think it should be added to the folder.

*This script is a part of Python notebook is a part of ‘example/model_update/4_update_generic_model_with_mri_data.ipynb’ in the repository. We added an explanation in the text:*

*L. 509-512: “Wrapping surface radii, length, and orientation were preserved from the generic-scaled model. During adjustment, a custom Python script, a part of the “4_update_generic_model_with_mri_data.ipynb ” notebook, verified potential intersections between muscle path points and wrapping surfaces to ensure accurate alignment”.*

>Lines 421-423: Adjustment boundaries were customised for different muscles [...].

Comment: Please explain why, and how the boundaries were defined.

*Thank you for this comment. We did not provide a sufficient explanation here. The current text says L. 537-539: “Adjustment boundaries were customised for different muscles. For example, gluteus maximus via points could move within a 2 cm radius, while rectus femoris and vastii points at the patella were limited to a 0.5 cm radius.” The rationale behind boundary choices depends on the size of the muscle in the vicinity of the point. If the muscle is represented by a thin tendon, such as patella tendon, the search was limited to 0.5 mm in each direction. If the muscle had a very large cross-section in the vicinity of via points, such as, for example, gluteal maximus via points, the search was extended to 2 mm in each direction.*

*The appropriate explanation has been added to the text L. 539-542: “The radius for each search space was determined by the muscle cross-sectional area in the vicinity of the point. For muscles represented by thin tendons, such as the patellar tendon, the search was limited to a smaller radius than for muscles with large cross-sectional areas like gluteus maximus.”*

>Lines 447-448: Furthermore, model validation was performed by comparing muscle activations estimated by static optimisation with EMG data collected from 16

muscles.

Comment: Could the authors please add some more details on the implementation of the static optimisation? Was it a weighted optimisation or the traditional equation?

*We expanded information about static optimisation on L.588-589 “static optimization (minimizing the sum of squared muscle activations with equal weighting).”*

- Discussion -

>Lines 231-236: [...] sensu Hickes et al.

Comment: Please amend. It should read Hicks et al.

*Thank you very much for pointing out this error. We changed the name.*

>Lines 231-236: [...] except for pelvis tilt.

Comment: Please discuss more about this point, which is not unexpected, but that was largely not commented on nor quantified in the Results section.

*Having removed the cosine comparison between stride vectors (at the request of the first reviewer), we no longer have the ground for discussing pelvis coordinates. We have, therefore, removed the discussion of the pelvis tilt from the text.*

>Lines 231-236: Joint moments also showed strong agreement, consistent with previous personalization studies

Comment: Please discuss on the impact of the inertial parameters that, from my understanding, were not updated in the MRI-based (vs generic scaled) models.

*We are not aware of publications addressing this aspect. However, the last author presented at an ISB conference approximately 10 years ago, exactly this investigation, and found negligible differences when personalizing the inertia properties based on MRI images.*

*We added a discussion of this aspect on L. 320-324: “This similarity likely stems from the use of identical anatomical coordinate systems and degrees of freedom in both models* [*[36]*](https://paperpile.com/c/09IS7w/uyn7y) *as well as from our choice to maintain musculoskeletal parameters and segment inertial properties in the MRI-based models the same as in the generic-scaled variants. Consequently, joint moments showed strong agreement between model types, consistent with previous personalization studies* [*[37]*](https://paperpile.com/c/09IS7w/RcIgW)*.”*

*on L.363-370. “Our study has several limitations. We did not account for individual motor control strategies; EMG-informed approaches may reduce excessive muscle activations* [*[50]*](https://paperpile.com/c/09IS7w/Nv8Kf)*. Morphological changes in personalized models may require corresponding muscle-tendon parameter adjustments* [*[51,52]*](https://paperpile.com/c/09IS7w/ob8Wm+fKh2Y)*. We did not modify segment inertial properties when creating MRI-based models, though we expect negligible impact. While we lack ground truth data for our participants, instrumented prosthesis studies* [*[11]*](https://paperpile.com/c/09IS7w/Ud5qF) *suggest personalized models produce more accurate results. Despite these limitations, our findings demonstrate that personalization affects biomechanical outcomes even in healthy populations.”*

*And on L. 543-547: “We chose to keep musculotendon parameters (tendon slack length and optimal fiber length) and segment inertial properties in the MRI-based models the same as in the generic-scaled models to ensure that any observed differences between the two model types were due to geometry only. Initially, we did not expect to obtain noticeable differences in limb segment lengths between the two model types.”*

>Lines 248-249: Overall, MRI-based models exhibited greater variability in joint contact forces than generic scaled models, with estimates for some individuals reaching

up to 6.5 body weights

Comment: Please report the mean and standard deviation when using MRI-based vs generic scaled models. It would be more informative and would better support the

sentence.

*This has been added to the manuscript, both in the abstract (L. 43-44) and in the results L. 251-254: “During early stance, hip contact force (Hip Peak 1) was 0.73±0.85 body weight (BW) higher in MRI-based models, while knee contact force (Knee Peak 1) was 0.84±1.28 BW higher. Other peaks showed smaller differences between models (Fig. 4b). Notably, the magnitude of these differences varied considerably across individuals (Fig. S4).”*

>Lines 283-285.

Comment: I agree, but anatomy is not the only important aspect in musculoskeletal models. Especially when one is interested in predicting muscle forces and joint contact forces. In fact, these quantities are largely affected by how muscles work, which depends on the internal muscle parameters too.

*This is a valid argument. In this paper, we chose to concentrate on the effect of geometry. However, muscle parameters optimization or personalization are very important when one aims to obtain realistic muscle forces. We added this limitation of the study in L.364-371.*

Comment: Last, I believe that the Discussion section should report on the time expenditure and – if performed – on the operator dependency.

*We have added appropriate information in Methods, L.433-434:*

*“An experienced operator requires approximately 2 hours to create an MRI-based model using this pipeline.”*

******************************************************************************************************

Reviewer #3:

This paper demonstrates the impact of model personalization using a semi-automated MRI-based method on segment geometries and joint kinematics/kinetics. Model personalization is a key area of importance to improve musculoskeletal modelling estimates and accessibility of workflows is a major limiting factor that requires addressing. For these reasons, I believe this paper provides a useful addition to the literature.

**Title**

1. Reconsider the question in the title. The general answer is yes. The nuanced answer depends on the intended use. Either way, the study is likely unable to answer the question.

*Thank you for the comment. We agree with your statement. Following this and other comments below, we made sure to qualify our recommendation to use MRI-personalised morphology for predicting joint contact forces.*

*L. 371-376*: *“The model choice depends on the research question. Generic-scaled models may be sufficient for general kinematics-driven analyses, e.g., comparing joint contact forces between walking and running. However, imaging-based personalization becomes necessary when addressing anatomy-specific questions, e.g., how femoral anteversion differs between sexes and how this affects joint contact forces. Our paper highlights uncertainties that should be considered when using generic-scaled models.”*

**Abstract**

2. Consider including high-level summary results (e.g. X% difference)

*Thank you for the suggestion. We have added the resulting numbers in the abstract.*

*L. 35-47: “The results revealed clear geometric differences between the model types, with MRI-based models exhibiting a wider pelvis (mean distance between ischial bones was 98.0*$\pm$*5.0 mm in generic-scaled and 11.0*$\pm$ *8.0 mm in MRI-based models) and distinct femur/tibia proportions (the mean ratio was 0.92*$\pm$*0.040 in generic-scaled and 0.99*$\pm$*0.033 in MRI-based models ). MRI-based models captured systematic anatomical differences between males and females that were absent in generic-scaled models. These geometric differences substantially affected joint loading estimates. MRI-based models consistently produced higher peak joint contact forces with greater inter-individual variation, particularly at the knee joints. Early stance knee peak joint contact force was higher in the MRI-based compared with the generic-scaled model by 0.84±1.28 body weights on average. Despite these differences in geometry and loading, joint kinematics were similar within individuals (mean difference was 0.8*$\pm$ $2.47^{\circ}$*) and muscle moment arms aligned well with published cadaver data, supporting the validity of the personalization approach. “*

**Introduction**

3. Line 110 – “and focuses on creating fully personalized musculoskeletal models for biomechanical simulations.” – Replace fully with skeletal anatomy only.

*We are personalizing muscle paths as well as joint center locations. We added appropriate explanations in the abstract and in the Introduction.*

*L. 26-28: “Our workflow concentrates on creating subject-specific joint centers and muscle paths. It also reconstructs bone surfaces without requiring MRI segmentation”.*

4. Current aim states outcomes as joint angle, joint moments, and joint contact forces. Results also include segment dimension and comparison of male and female.

*We have changed the sentence to a more general statement that encompasses all results*

*L. 32-34: “Two model types were compared using a number of parameters, including model geometry, joint kinematics, dynamics, and resultant joint contact forces during one walking cycle.”*

*L. 146-150 “To evaluate this workflow and challenge the assumption that personalization is unnecessary for healthy adults, we compare MRI-based musculoskeletal models with generic-scaled models in eight healthy adults. We examined the impact of personalization using a number of parameters, including muscle moment arms, joint kinematics, joint moments, and joint contact forces during walking*.”

5. Line 115 – “By addressing the assumption that personalization is unnecessary for healthy adults, we demonstrate the importance of accounting for individual anatomical variability, even in populations where generic models are often considered sufficient.” – I think it is difficult to make statements about whether personalisation is “necessary” or not, because it is dependent on the question. For some applications, a generic model may be sufficient. How you may want to frame it is that personalisation has a significant impact on the estimation of joint/muscle forces. Therefore, if the question requires an accurate “true” value, personalisation is likely important.

*We have reworded this sentence, L. 48-51: “This workflow simplifies the creation of MRI-based musculoskeletal models and challenges the assumption that personalization is unnecessary for healthy adults. The findings reveal significant sensitivity of joint contact forces to individual morphology, emphasizing the importance of personalized models even in healthy populations for biomechanical analyses.”*

**Results**

6. Is there a reason for using cosine similarity rather than RMSD?

*Thank you for this question. The cosine similarity allows for comparison of vector quantities. Normalised gait cycle waveforms are such vectors. However, we recognise that cosine similarity is not frequently utilised in gait analysis. Therefore, we now report averages and standard deviations.*

7. Each outcome variable uses a different comparison approach (% difference, RMSD, cosine similarity, and plotting boxplots side-by-side). This inconsistency makes a relatively simple set of comparisons more challenging to assess.

*We have now streamlined results reporting and provide mostly averages and standard deviations for linear measurements and Euclidean distances for landmark locations in 3D.*

8. For validation, I recommend reporting the actual values (mean and SD) for error/tracking broken down by MRI-based and generic models.

*We have now reported means and standard deviations*.

9. Consider presenting the moment arms in the main text. This will help capture the direct impact of anatomical and muscle path changes.

*We have created an additional figure (Supplementary information Figure S1), which compares muscle via points and joint centres in the round frame and displays moment arms for selected muscles.*

10. The correlation analysis doesn’t seem to add much value. It is unclear which geometrical characteristics are captured by each PC and, therefore, the interpretation of why that would explain a correlation with joint contact force.

*Thank you for raising this question. PCA is a very popular way to summarise shape differences (Slice, 2005; Zeldich et al., 2012). We understand that the reviewer did not find the figure with the shape differences described by the first principal component to be self-explanatory. We re-worked the figure (now Figure 2) and expanded the description of the meaning of the first principal components in the text***.**

*L. 209-221:* *“The first two principal components (PCs) described 63.9% of the variance (Fig. 2a). PC1 (51.0% of variance) strongly differentiated between MRI-based and generic-scaled models with no overlap between the two groups. For the pelvis, this component differentiated between shapes with a narrow distance between the ischial bones and a relatively posterior position of the torso origin, as in the generic-scaled models, from those with a relatively wider distance between ischiopubic arches, wider distance between hip joint centers and a relatively anterior position of the torso origin, as in the MRI-based models (Fig. 2b). For the limb segments, this component differentiated models with a relatively shorter femur and a longer tibia, as in the generic-scaled models, from those with a relatively longer femur and a shorter tibia, as in the MRI-based models (Fig. 2b). PC2 (12.0% of variance) differentiated MRI-based male and female models, where females tended to have a wider pelvis and a relatively shorted femur than males in our study cohort (Fig. 2c). Generic-scaled models did not differentiate between males and females”.*

*The correlation analysis demonstrates the tendency of the generic-scaled morphology to associate with lower values of the joint contact forces (L. 265-267): “To investigate the general trend in the relationship between joint contact forces and bone morphology, we computed Pearson's correlations between peak joint contact forces and the first principal component (PC1), which differentiates between MRI-based and generic-scaled models based on pelvis, femora, patellae, and tibiae shape (Fig.5a)”.*

**Methods**

11. Line 331 “activation signals were collected on the left and right lower limb with the help of EMG sensors.” – Consider using the term muscle excitation. “Help” is not the best word to use.

*Thank you. We have changed the word in L.410-413 “Gluteus maximus, gluteus medius, tensor fasciae latae, biceps femoris, rectus femoris, vastus lateralis, vastus medialis, and gastrocnemius medialis activation signals were collected on the left and right lower limbs using EMG sensors.”*

12. Line 343 “MRI-based models featured joint centres in MRI according to ISB definitions and muscle paths closely matching actual anatomy.” – Why only the MRI-based model, do both models not follow the ISB joint centre definitions? What is meant by the statement on muscle paths? Is this referring to the base model or the personalised model?

*Thank you for this important clarification. You are correct that both model types follow ISB joint center definitions. We have revised the text to clarify that the key distinction is how these definitions are applied: generic-scaled models use motion capture marker-based anthropometric scaling, while MRI-based models define joint centers directly from MRI anatomical landmarks and adjust muscle via points to individual bone geometry.*

*L. 423-429: “We used the Rajagopal et al.* [*[33]*](https://paperpile.com/c/09IS7w/Nra5D/?noauthor=1) *model, a commonly used OpenSim model with arms removed and torso mass adjusted. For each participant, we created both a generic-scaled model (standard scaling from motion capture markers) and an MRI-based model (joint centers from MRI landmarks, muscle via points adjusted to individual geometry). Both followed ISB conventions. Bone surfaces of the MRI-based models were approximated for display purposes only.”*

13. Line 366 “included updating the generic-scaled model with muscle paths” – This is the first mention that the pipeline includes muscle path personalisation. Obviously, changing bone dimensions will change muscle paths, but as I understand it, the muscle paths themselves are not being personalised (e.g., by tracking the shape of muscle volume or adjusting attachment sites).

We have now added explicit mention of muscle paths personalization in the abstract and in the Introduction.

L 26-28: “*Our workflow concentrates on creating subject-specific joint centers and muscle paths. It also reconstructs bone surfaces without requiring MRI segmentation.*”

14. Line 391 “Next, the data were separated into segments” – Please specify what data

*We added an explanation in the sentence L. 496-497:*

*“Next, the landmark data, i.e. 3D coordinates of joint locations, muscle paths and skin markers, were separated into segments (folder/example/model_update/2.0_use_mri_data.ipynb).”*

**Conclusion**

15. “non-linear approach” only in the discussion is the approach referred to as non-linear. What would a linear approach look like? I recommend sticking with terminology used previously (MRI-based personalisation of skeletal geometry).

*Thank you for this suggestion. We have removed 'non-linear' terminology from the Conclusions and consistently use 'MRI-based personalization' throughout (L 379-391)*

16. Line 299 “Our findings challenge the assumption that personalization is unnecessary for healthy adults, demonstrating that even in this population, generic models fail to capture important anatomical differences that influence simulation outcomes.” – As per previous comment, I think it is well understood that generic models do not capture anatomical differences. The point to make is that personalisation has substantial impacts, and therefore, in applications where accuracy is important, personalisation should be considered.

*Thank you for this clarification. We have revised the Conclusions (L 379-391) to emphasize that personalization substantially impacts joint contact force estimations in healthy adults, rather than simply noting that generic models don't capture anatomical variation. The revised text now focuses on when personalization should be considered based on the research question and required accuracy.*

17. Line 303 “Unlike previous non-linear personalization methods, our workflow does not require large population samples to predict muscle paths.” – What methods requiring large samples are you referring to?

*Thank you for this point. We have now expanded the review of relevant methods in the Introduction L. 110-130.*

**Github repository** (optional feedback)

*Тhank you for your suggestions for improving the repository. We are grateful for your input and happy to follow up on some of the suggestions. We place responses directly below.*

- Consider renaming the repository. It isn’t clear what TPS means without reading the paper.

*We have changed the name of the repository to:*

*https://github.com/katya-stanzy/thin-plate-spline_personalised_muscoloskeletal_model.git*

- Overall, the instructions are ok, and the diagrams are very helpful. But there are many areas that could be improved to make it more intuitive and easier to follow.
  - GitHub instructions could be clearer and step-by-step
- *The nature of the process is such that many of the directions are local to particular notebooks and do not translate well to a general set of instructions.*
  - Organization of folders in repo
  - Naming of scripts & files
- *Unfortunately, we are not able to change the organisation of folders and naming of scripts and files at this stage, as it would require making a substantial amount of changes due to the file cross-reference in the notebooks.*
- Consider having one complete set of instructions rather than following some instructions in the readme and some within the notebooks

*The nature of the process is such that many of the directions are local to particular notebooks and do not translate well to a general set of instructions.*

- Consider adding steps for setting up VSCode & Slicer with Jupyter

*Appropriate references have been added in README.md*

- Limited instructions on how to place markers in Slicer 3D for those with no experience

*Appropriate references have been added in README.md*

- Provide more instruction on loading/saving data from Slicer 3D. I find there are a couple of ways to do this and different file type options.

*Appropriate references have been added in* [*README.md*](http://readme.md) *:*

*”3D Slicer project that you will have created needs to be saved via File -> Save Data -> "Save Scene and Unsaved Data" window. Please do not create a zip folder for the project, as its files will have to be accessible for Jupyter IDE.”*

- Under examples, DF0109 seems to be missing data, AM12 worked.

*We do not recognise these filenames: they do not appear in our repository.*

CITATIONS

[Bookstein, Fred L. 1991. “Thin-Plate Splines and the Atlas Problem for Biomedical Images.” In *Information Processing in Medical Imaging*, edited by Alan C. F. Colchester and David J. Hawkes. Lecture Notes in Computer Science. Springer-Verlag.](http://paperpile.com/b/rhk06q/72cxe)

[Boyd, S. K., J. L. Ronsky, D. D. Lichti, K. Salkauskas, and M. A. Chapman. 1999. “Joint Surface Modeling with Thin-Plate Splines.” *Journal of Biomechanical Engineering* 121 (5): 525–532.](http://paperpile.com/b/rhk06q/UpzPb)

[Slice, D. E. 2005. “Modern Morphometrics.” *Modern Morphometrics in Physical Anthropology*.](http://paperpile.com/b/rhk06q/vwTEl) <https://link.springer.com/chapter/10.1007/0-387-27614-9_1>[.](http://paperpile.com/b/rhk06q/vwTEl)

[Zelditch, Miriam Leah, Donald L. Swiderski, and H. David Sheets. 2012. *Geometric Morphometrics for Biologists: A Primer*. 2nd ed. Academic Press.](http://paperpile.com/b/rhk06q/5sEuN) <https://sbmorphometrics.org/books/macleod_review_2005.pdf>[.](http://paperpile.com/b/rhk06q/5sEuN)
